# High-Throughput Sequencing With the Preselection of Markers Is a Good Alternative to SNP Chips for Genomic Prediction in Broilers

**DOI:** 10.3389/fgene.2020.00108

**Published:** 2020-02-27

**Authors:** Tianfei Liu, Chenglong Luo, Jie Ma, Yan Wang, Dingming Shu, Guosheng Su, Hao Qu

**Affiliations:** ^1^State Key Laboratory of Livestock and Poultry Breeding, Institute of Animal Science, Guangdong Academy of Agricultural Sciences, Guangzhou, China; ^2^Guangdong Provincial Key Laboratory of Animal Breeding and Nutrition, Institute of Animal Science, Guangdong Academy of Agricultural Sciences, Guangzhou, China; ^3^Center for Quantitative Genetics and Genomics, Department of Molecular Biology and Genetics, Aarhus University, Tjele, Denmark

**Keywords:** genomic prediction, high-throughput sequencing, marker screening method, feed conversion ratio, chickens

## Abstract

The choice of a genetic marker genotyping platform is important for genomic prediction in livestock and poultry. High-throughput sequencing can produce more genetic markers, but the genotype quality is lower than that obtained with single nucleotide polymorphism (SNP) chips. The aim of this study was to compare the accuracy of genomic prediction between high-throughput sequencing and SNP chips in broilers. In this study, we developed a new SNP marker screening method, the pre-marker-selection (PMS) method, to determine whether an SNP marker can be used for genomic prediction. We also compared a method which preselection marker based results from genome-wide association studies (GWAS). With the two methods, we analysed body weight at the12^th^ week (BW) and feed conversion ratio (FCR) in a local broiler population. A total of 395 birds were selected from the F2 generation of the population, and 10X specific-locus amplified fragment sequencing (SLAF-seq) and the Illumina Chicken 60K SNP Beadchip were used for genotyping. The genomic best linear unbiased prediction method (GBLUP) was used to predict the genomic breeding values. The accuracy of genomic prediction was validated by the leave-one-out cross-validation method. Without SNP marker screening, the accuracies of the genomic estimated breeding value (GEBV) of BW and FCR were 0.509 and 0.249, respectively, when using SLAF-seq, and the accuracies were 0.516 and 0.232, respectively, when using the SNP chip. With SNP marker screening by the PMS method, the accuracies of GEBV of the two traits were 0.671 and 0.499, respectively, when using SLAF-seq, and 0.605 and 0.422, respectively, when using the SNP chip. Our SNP marker screening method led to an increase of prediction accuracy by 0.089–0.250. With SNP marker screening by the GWAS method, the accuracies of genomic prediction for the two traits were also improved, but the gains of accuracy were less than the gains with PMS method for all traits. The results from this study indicate that our PMS method can improve the accuracy of GEBV, and that more accurate genomic prediction can be obtained from an increased number of genomic markers when using high-throughput sequencing in local broiler populations. Due to its lower genotyping cost, high-throughput sequencing could be a good alternative to SNP chips for genomic prediction in breeding programmes of local broiler populations.

## Introduction

Genomic prediction is a new generation breeding technology, and it has been widely implemented in animal and plant breeding (Meuwissen et al., 2001; Su et al., 2016; Wang et al., 2019). Genomic prediction, which uses information from markers throughout the whole genome, can achieve accurate early selection, especially for those traits that are difficult or costly to measure, such as sex-limited traits and slaughter traits. Many studies have shown the advantages of genomic prediction in poultry (Sitzenstock et al., 2013; Wolc et al., 2013; Wolc et al., 2015; Zhang et al., 2017; Liu et al., 2019). However, the high cost of genome marker genotyping limits the application of genomic prediction in poultry. With the development of low-cost and high-throughput sequencing, various marker genotyping platforms have provided alternatives to chip-based genotyping.

The choice of marker genotyping platform is a key factor affecting the accuracy of genomic estimated breeding values (GEBV) (Tan et al., 2017; Wang et al., 2019; Whalen et al., 2019). Single nucleotide polymorphism (SNP) chips are currently the most common choice in livestock and poultry (Wolc et al., 2011; Wang et al., 2013; Zhang et al., 2017). Recently, with the development of high-throughput sequencing technology, reduced-representation genome sequencing (RRGS) has been developed. RRGS uses restriction endonucleases to digest genomic DNA and then sequence the digested fragments, such as restriction site associated DNA (RAD) (Baird et al., 2008), genotyping-by-sequencing (GBS) (Elshire et al., 2011; Wang et al., 2017), and specific-locus amplified fragment sequencing (SLAF-seq) (Sun et al., 2013).

RRGS can produce a large number of genomic markers at a low price and thus be used as an alternative genotyping platform, but the genotype quality of the markers is lower than that obtained with SNP chips (Gorjanc et al., 2015). In animal breeding, the application of RRGS in genomic prediction has attracted great attention and led to many studies (Tan et al., 2017; Wang et al., 2019; Whalen et al., 2019). Gorjanc et al. (Gorjanc et al., 2015) used simulation data to show that the use of GBS for genotyping has great potential for genomic selection in livestock populations. Tan et al. (2017) used GBS for genotyping in a Duroc boar population, and the accuracy of genomic prediction for teat number was 0.435, but this study did not compare their results to those from SNP chips.

There are many local broiler poultry breeds with unique characteristics around the world, such as Chinese yellow feather chickens and French Label Rouge chickens. Compared to SNP chips, a sequencing approach can obtain genetic variations specific to local breeds and, thus, may achieve higher accuracy than SNP chips.

The accuracy of genomic prediction does not increase dramatically as the number of markers increases exponentially (Heidaritabar et al., 2016; Ni et al., 2017). Heidaritabar et al. (Heidaritabar et al., 2016) compared the differences of the accuracy of genomic prediction for the number of eggs between whole genome sequencing data and 60K gene chip data in a commercial white layers line. It was found that the accuracy of genomic prediction from the sequencing data was only increased by ~1%. Most of the important economic traits are quantitative traits that are controlled by multiple genes. We believe that not all SNP sites have an effect on traits, and the number of markers that affect traits is limited. Thus, sequencing data can increase the number of associated markers and introduces a large number of unimportant markers that are not related to the traits of interest, which interferes with the estimation of breeding values. Therefore, selecting effective markers for genomic prediction is expected to improve the prediction accuracy. How to select markers from high-throughput sequencing data for genomic prediction is an important issue.

The aim of this study was to: (1) propose a novel method to screen markers for genomic prediction, and (2) compare the accuracy of genomic prediction between high-throughput sequencing and SNP chips in broilers.

## Materials and Methods

### Data

The broiler population used in the current study was established by crossing the “High Quality chicken Line A” (HQLA) with the Huiyang Beard chicken (HB)(Sheng et al., 2013). The HQLA line has been under selection for growth traits and high meat quality tailored to Chinese tastes for more than 10 generations. The HB line is a Chinese indigenous breed with the characteristics of slow growth and high meat quality. In this study, 395 individuals (212♂+ 183♀) were selected from 8 half-sib families of 511 F2 birds, which originated from 20 F0 ancestors (6♂+ 14♀), and GBS with 10X specific-locus amplified fragment sequencing (SLAF-seq) (Sun et al., 2013) and the Illumina Chicken 60K SNP Beadchip (Groenen et al., 2011) were used for genotyping. Twenty-eight autosomes and a sex chromosome (chrZ) were extracted for the further analyses. To ensure the integrity of the SNP marker coverage in the sequencing data, only the markers covering more than 70% of the genotype were retained. Then, the marker data were edited by deleting markers with a minor allele frequency (MAF) lower than 0.01. After quality control, 121,132 SLAF-seq markers and 46,690 chip markers were obtained, respectively. Missing genotypes were ignored for preselection of markers and were replaced by the expected genotype score (i.e. 0 after centering) for Gmatrix program, and the average number of missing genotypes per individual were 6,233 and 232 for the SLAF-seq and chip data, respectively.

As shown in **Table 1**, two of the most important traits in broilers were analysed, namely body weight at the 12^th^ week (BW) and the feed conversion ratio (FCR). Body weight and feed intake were measured during the period from the beginning of the 7^th^ to the end of the 12^th^ week (42 d). FCR was calculated as the ratio of average daily feed intake to average daily gain, as described in Liu et al. (2017). Corrected phenotypic values (y_c_), instead of original observations (y), were used as response variables to calculate the difference between phenotypes of the two homozygous genotypes and to predict breeding values using SNP markers. The reason for using y_c_ as response variables was to reduce noise by removing fixed effects which could be estimated much more accurately using a larger dataset, rather than using only genotyped animals with the two genotyping platforms. The fixed effects were estimated using linear least squares regression including sex (two levels) and batch (six levels), and y_c_ = y – sex effect – batch effect.

**Table 1:** Number of observations (N), mean, standard deviation (SD), minimum value (Min), and maximum value (Max) for body weight (BW) at 12^th^ week and feed conversion ratio (FCR) during the period from 7^th^ to 12^th^ week.

Trait	N	Mean	SD	Min	Max
BW	395	2034	360	927	3250
FCR	390	3.56	0.39	2.83	6.29

### Statistical Models

#### SNP Marker Screening Method

In this study, we provide a new method, the pre-marker-selection (PMS) method, to screen informative markers for genomic prediction based on the difference between phenotypes of the two homozygous genotypes at the marker with the data of the reference population, and the marks which have no homozygote or only have one homozygote will be deleted. The model is

$$d=|{\bar{x}}_{A_{1}A_{1}}-{\bar{x}}_{A_{2}A_{2}}|,$$

where d is absolute value of the difference between phenotypes of the two homozygous genotypes A_1_A_1_ and A_2_A_2_; ${\bar{x}}_{A_{1}A_{1}}$and ${\bar{x}}_{A_{2}A_{2}}$ are the mean of corrected phenotypic values of the genotypes A_1_A_1_ and A_2_A_2_, respectively.

To generalize the PMS method, a transformation was applied, *d*′ = d/max (*d*). After transformation, *d* ′ was in the interval [0,1]. To explore the suitable cutoff value with PMS method, four different *d* ′ value cutoffs (0.001, 0.01, 0.05, 0.1) were set to prune markers of genome data, the peak value of genomic prediction accuracy were on the cutoff value of 0.05, the summary of this preselection is presented in **Tables 2** and **3**. In the current study, all SNP markers with *d′* greater than 0.05 were retained in leave-one-out cross-validation method.

**Table 2:** No. of single nucleotide polymorphism (SNP) markers after preselection using the premarker-selection (PMS) method with different cutoff values.

Cutoff Value^1^	Seq-BW^2^	Chip-BW	Seq-FCR	Chip-FCR
0	121,132	46,690	12,1132	46,690
0.001	113,423	36,598	111,550	36,423
0.01	98,380	32,333	80,450	30,892
0.05	43,959	15,748	10,976	11,506
0.1	13,423	5,000	1,706	3,118
0.2	2,689	930	202	527

^1^Cutoff value of 0 indicates the scenario of using all SNP markers without preselection, and the others were cutoff values indicate the use of the PMS method.

^2^BW , body weight at the 12^th^ week, FCR , feed conversion ratio; Seq, High-throughput sequencing markers; Chip, Chip markers.

**Table 3:** Accuracy of genomic prediction using the markers preselected by premarker-selection (PMS) method with different cutoff values.

Cutoff Value^1^	Seq-BW^2^	Chip-BW	Seq-FCR	Chip-FCR
0	0.509	0.516	0.247	0.231
0.001	0.515	0.520	0.263	0.243
0.01	0.558	0.538	0.380	0.296
0.05	0.674	0.607	0.496	0.422
0.1	0.697	0.628	0.377	0.416
0.2	0.577	0.535	0.279	0.273

^1^Cutoff value of 0 indicates the scenario of using all single nucleotide polymorphism (SNP) markers without preselection, and the others were cutoff values indicate the use of the PMS method.

^2^BW, body weight at the 12^th^ week; FCR, feed conversion ratio; Seq, High-throughput sequencing markers; Chip, Chip markers.

In this study, genome-wide association studies (GWAS) using single marker regression was also used as a control method to preselect marker. The markers were selected based on the *p* values from GWAS results with the data of the reference population. The GWAS model is

y=1μ+xq+ε

where **y** is the vector of corrected phenotypic values of body weight at the 12^th^ week and FCR, μ is the intercept, q is the effect of the marker in the model, which is treated as a fixed regression of observation on genotype, **x** is a vector containing genotypes of the marker with 0 for A_1_A_1_, 1 for A_1_A_2_ and 2 for A_2_A_2_, and **ε** is a vector of random deviates, which is assumed that $\varepsilon \sim \text{N}\left(0,I\sigma_{\varepsilon }^{2}\right)$

To explore the suitable cutoff value with GWAS method, three different cutoff *p* values (0.001, 0.01, 0.1) were set to prune markers of genome data, the peak value of genomic prediction were on the cutoff value of 0.01, the summary of this preselection is presented in **Tables 4** and **5**. In the current study, all SNP markers with *p* value less than 0.01 were retained in leave-one-out cross-validation method.

**Table 4:** No. of single nucleotide polymorphism (SNP) markers with preselection using genome-wide association studies (GWAS) method for preevaluation the cutoff value.

Cutoff Value^1^	Seq-BW^2^	Chip-BW	Seq-FCR	Chip-FCR
1	12,1132	46,690	12,1132	46,690
0.1	7,647	13,401	5,439	2,362
0.01	1,998	817	1,742	345
0.001	1,252	133	1,316	111

^1^Cutoff value of 1 indicates the scenario of using all SNP markers without preselection.

^2^BW, body weight at the 12^th^ week, FCR, feed conversion ratio; Seq, High-throughput sequencing markers; Chip, Chip markers.

**Table 5:** Accuracy of genomic prediction using the markers preselected by genome-wide association studies (GWAS) method with different cutoff values.

Cutoff Value^1^	Seq-BW^2^	Chip-BW	Seq-FCR	Chip-FCR
1	0.509	0.516	0.247	0.231
0.1	0.566	0.561	0.357	0.419
0.01	0.573	0.596	0.345	0.449
0.001	0.487	0.545	0.249	0.395

^1^Cutoff value of 1 indicates the scenario of using all single nucleotide polymorphism (SNP) markers without preselection.

^2^BW, body weight at the 12^th^ week, FCR, feed conversion ratio; Seq, High-throughput sequencing markers; Chip, Chip markers.

#### Genomic Prediction Model

In the current study, breeding values were estimated using a genomic best linear unbiased prediction model (GBLUP). The GBLUP model is

y=1μ+Zg+e

where the definitions of **y** and μ are the same as above, **g** is the vector of genomic breeding values to be estimated, **Z** is the incidence matrix of **g**, and **e** is the vector of random residuals. It is assumed that $g\sim \text{N}\left(0,G\sigma_{g}^{2}\right)$ and $e\sim \text{N}\left(0,I\sigma_{e}^{2}\right)$, where **G** is the additive genomic relationship matrix based on SNP markers (Vanraden, 2008)**, *G***=***MM***′/Σ2*p*_*i*_(1−*p*_*i*_), the coefficients of the i^th^ column in the **M** matrix are (0–2p_i_) for genotype A_1_A_1_, (1–2p_i_) for A_1_A_2_, and (2–2p_i_) for A_2_A_2_, where p_i_ is the allele frequency of A_2_ at locus i, and $\sigma_{g}^{2}$ is the genomic additive genetic variance.

#### Cross-Validation Method

To eliminate all problems associated with the random partitioning variation with n-fold cross-validation, the accuracy of genomic prediction was verified by leave-one-out cross-validation method (Allen, 1971). All 395 individuals were used for pre-evaluation of the cutoff values of the SNP marker screening methods. For each leave-one-out validation, the used to preselect markers with PMS and GWAS methods were in line with the reference population of leave-one-out validation, i.e., the leave-out individual was excluded from the data for preselecting marker. For example, for BW, the validation repeated 395 times, and each time one bird was masked in preselection the SNP markers and then the bird was also masked in the leave-one-out cross-validation. In this study, the accuracy of prediction was defined as the correlation between the predicted and corrected phenotypic value (y_c_), the differences between the correlations were analysed by a bootstrapped paired t-test (Efron, 1979), the sample was repeat 1,000 times. Unbiasedness of the GEBV was measured using the regression of y_c_ on GEBV. The regression will not differ significantly from one if GEBV is an unbiased estimate of true breeding value (Su et al., 2012).

The **G** matrix was calculated using Gmatrix package (https://dmu.ghpc.au.dk/DMU). The variance and covariance components were estimated using restricted maximum likelihood (REML) based on the mixed() function of “mixedFunction.R”, and leave-one-out cross-validations were performed with “mixedCV.R”. The R codes “mixedFunction.R” and “mixedCV.R” can be downloaded from the website in (Xu, 2017). The bootstrapped paired t-tests were performed with sample() and t.test() functions in R (https://www.r-project.org/).

## Results

### Distribution of Genomic Markers in the SLAF-seq and SNP Chip

**Figure 1:**
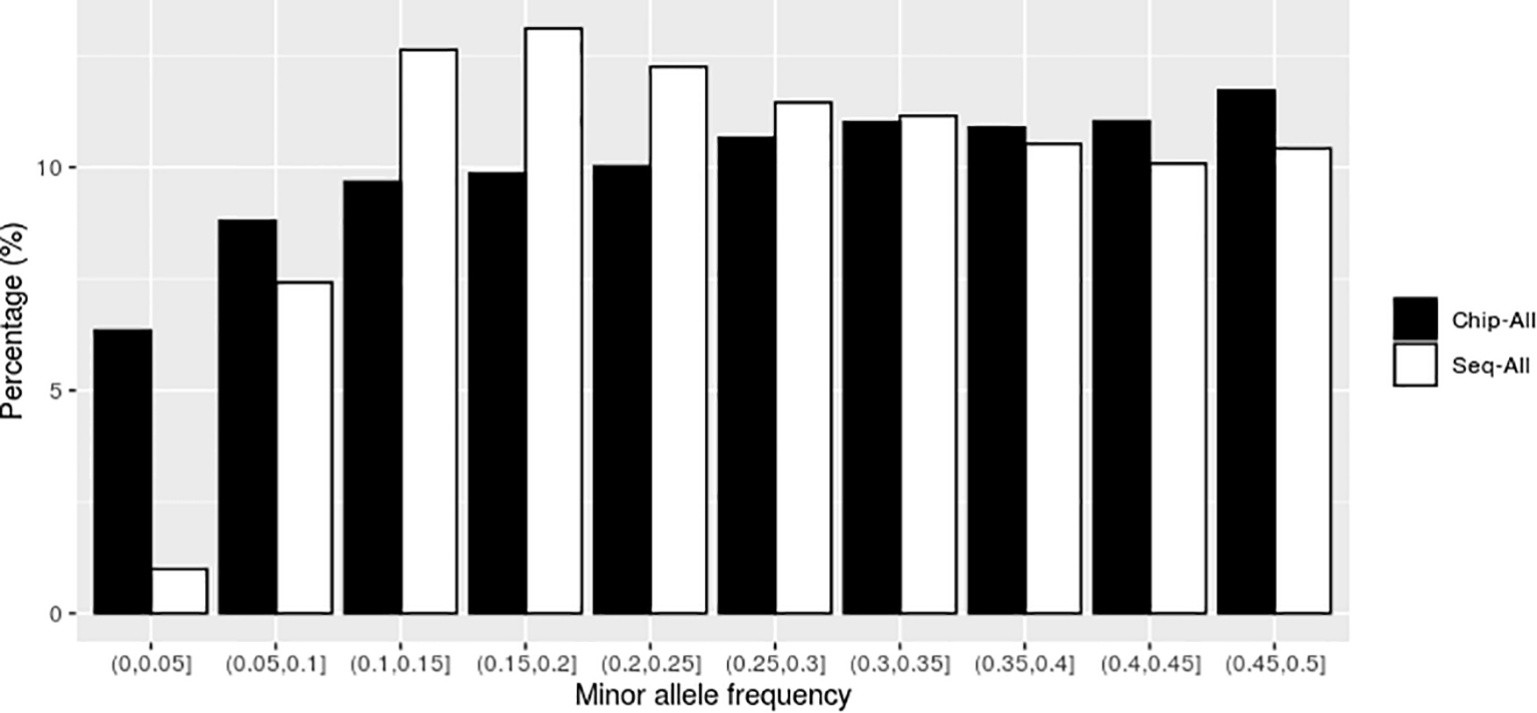
The distribution of genomic markers in the different intervals of minor allele frequency (MAF) without preselection. Chip-ALL, the scenario using all high-throughput sequencing markers without preselection; Sep-ALL, the scenario using all chip markers without preselection.

As shown in **Figure 1**, in the scenarios using all high-throughput sequencing markers (Sep-ALL) and all chip markers (Chip-ALL) without preselection, the uniformity of the number of genomic markers in MAF intervals was lower for SNPs obtained with SLAF-seq than for those obtained with the SNP chip. The coefficient of variation for the SNP markers in the MAF intervals, which was calculated as the ratio of the standard deviation to the mean, was 0.355 for SLAF-seq and 0.154 for the SNP chip.

All 395 individuals were used for assessing cutoff values in preselection of markers. As shown in **Tables 2** and **3**, with the PMS method, after unimportant markers were removed, the numbers of SNP markers obtained with the two genotyping platforms were both drastically reduced. The accuracies of genomic prediction with preselection of marker by all the cutoff values were higher than the accuracies using all markers. The peak values of genomic prediction accuracy were in the scenario with cutoff value of 0.1 for BW and 0.05 for FCR, respectively. As shown in **Tables 4** and **5**, with the GWAS method, after unimportant markers were removed, the number of SNP markers were greater reduced. The peak values of genomic prediction accuracy were in the scenario with cutoff value of 0.01 for the two traits.

**Figure 2:**
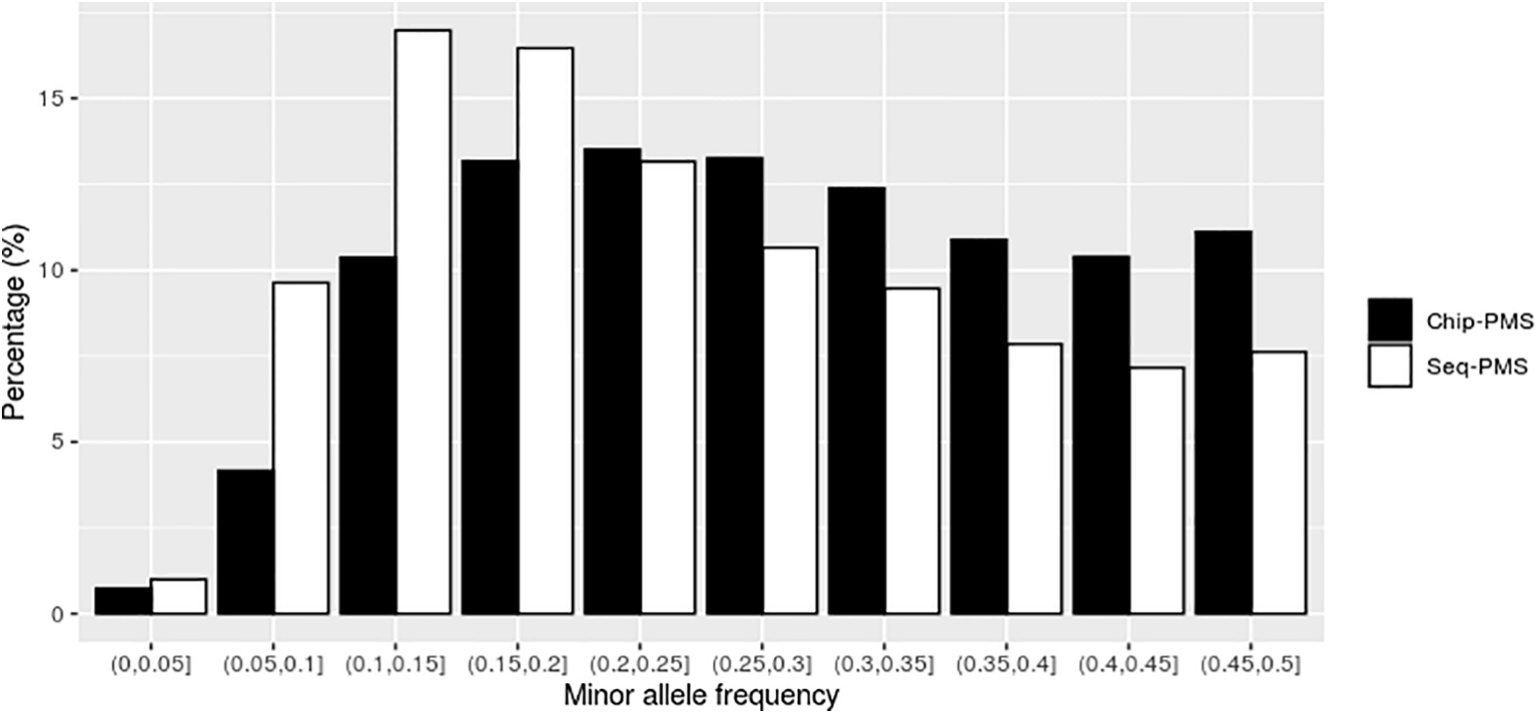
The distribution of genomic markers preselected by the premarker-selection (PMS) method for body weight in the different intervals of minor allele frequency (MAF). Chip-PMS, the scenario using high-throughput sequencing markers preselected by the PMS method; Sep-PMS, the scenario using chip markers preselected by the PMS method.

**Figure 3:**
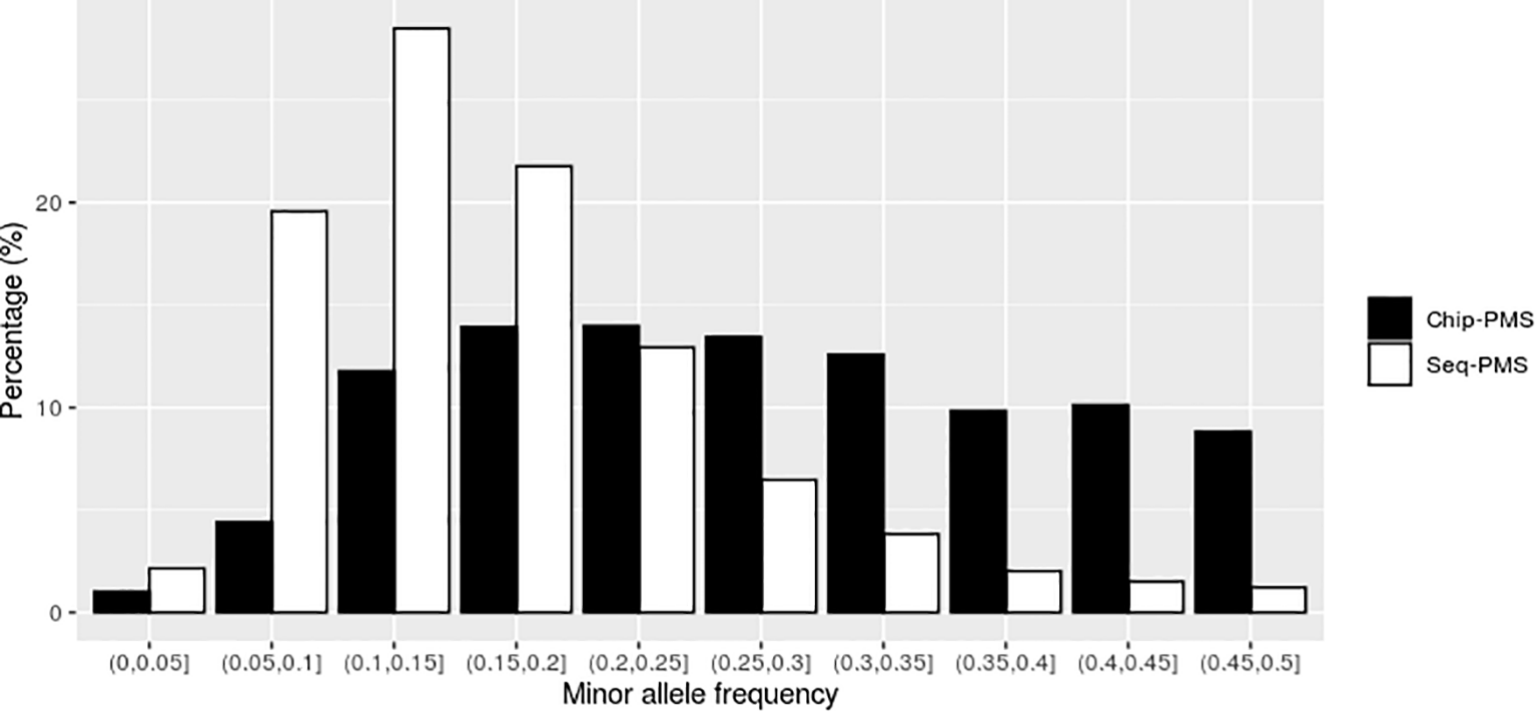
The distribution of genomic markers preselected by the premarker-selection (PMS) method for feed conversion ratio in the different intervals of minor allele frequency (MAF). Chip-PMS, the scenario using high-throughput sequencing markers preselected by the PMS method; Sep-PMS, the scenario using chip markers preselected by the PMS method.

Using SLAF-seq markers preselected by PMS method with best cutoff values, the number of SNPs was reduced from 121,132 to 13,423 for BW and 10,976 for FCR, respectively. As shown in **Figures 2** and **3**, the selected markers were mainly concentrated in the range of MAF from 0.05 to 0.25 for BW and FCR.

Using the SNP chip markers preselected by with PMS method with the best cutoff values, the number of markers was reduced from 46,690 to 15,748 and 11,506 for BW and FCR at the peak value of genomic prediction, respectively. As shown in **Figures 2** and **3**, the selected markers were mainly concentrated in the range of MAF from 0.1 to 0.3 for BW and FCR.

### Accuracy of Genomic Prediction Using the Two Genotyping Platforms

Without preselection of markers, the estimates of heritability for BW were 0.703 ± 0.087 using SLAF-seq and 0.634 ± 0.076 using the SNP chip. The estimates of heritability for FCR were 0.265 ± 0.099 and 0.266 ± 0.093, respectively.

As shown in **Table 6**, without marker screening, the genomic prediction model did not benefit from the increased number of genomic markers obtained by high-throughput sequencing, and the accuracy of genomic prediction using SLAF-seq was not always higher than the accuracy using the SNP chip. For BW, the accuracy of genomic prediction using SLAF-seq was lower than that using the SNP chip, and the difference between them was 0.007 (P < 0.05). For FCR, the accuracy of genomic prediction using sequencing was 0.017 higher than that using the gene chip (P < 0.05).

**Table 6:** Accuracy of genomic prediction for body weight and feed conversion ratio using markers preselected with the best cutoff value.

Scenario^1^	Seq-BW^2^	Chip-BW	Seq-FCR	Chip-FCR
S_PMS_	_a_0.671^A^ ± 0.035	_a_0.605^B^ ± 0.043	_a_0.499^A^ ± 0.035	_b_0.422^B^ ± 0.038
S_GWAS_	_b_0.606^A^ ± 0.037	_b_0.599^B^ ± 0.040	_b_0.428^B^ ± 0.033	_a_0.456^A^ ± 0.036
S_ALL_	_c_0.509^B^ ± 0.052	_c_0.516^A^ ± 0.053	_c_0.249^A^ ± 0.050	_c_0.232^B^ ± 0.050

^1^S_PMS_, the scenario using single nucleotide polymorphism (SNP) markers preselected by the premarker-selection (PMS) method with the cutoff value of 0.05; S_GWAS_, the scenario using SNP markers preselected by the GWAS method with the cutoff value of 0.01; S_ALL_, the scenario using all SNP markers without preselection.

^2^BW, body weight at the 12^th^ week, FCR, feed conversion ratio; Seq, High-throughput sequencing markers; Chip, Chip markers.

^A-B^Within a row, estimates without a common capital letters of the superscript for the same trait differ significantly (P < 0.05) according to the bootstrapped paired t-test.

^a-c^Within a column, estimates without a common lowcase letters of subscript differ significantly (P < 0.05) according to the bootstrapped paired t-test.

With the PMS method, the accuracy of genomic prediction for the two traits was improved, regardless of whether SLAF-seq or the SNP chip was used. When using SLAF-seq, the accuracy of genomic prediction was increased by 0.162 for BW and 0.250 for FCR, and the gains were significant (P < 0.05). Using the SNP chip, the accuracy of genomic prediction was increased by 0.089 for BW and 0.190 for FCR and the gains were also significant (P < 0.05). In addition, the accuracy of genomic prediction using SLAF-seq was higher than that using the SNP-chip by 0.066 for BW and 0.077 for FCR. As shown in **Table 7**, when the PMS method was applied, the regression coefficients for the two genotyping platforms were similar, genomic predictions for FCR had a slightly larger bias than those for BW.

**Table 7:** Unbiasedness of genomic prediction for body weight and feed conversion ratio using markers preselected with the best cutoff value.

Scenario^1^	Seq-BW^2^	Chip-BW	Seq-FCR	Chip-FCR
S_PMS_	1.046 ± 0.060	1.002 ± 0.074	0.948 ± 0.143	0.939 ± 0.128
S_GWAS_	0.994 ± 0.067	0.980 ± 0.073	0.896 ± 0.145	0.907 ± 0.130
S_ALL_	1.025 ± 0.100	1.001 ± 0.100	1.015 ± 0.181	0.926 ± 0.169

^1^S_PMS_, the scenario using single nucleotide polymorphism (SNP) markers preselected by the premarker-selection (PMS) method with the cutoff value of 0.05; S_GWAS_, the scenario using SNP markers preselected by the genome-wide association studies (GWAS) method with the cutoff value of 0.01; S_ALL_, the scenario using all SNP markers without preselection.

^2^BW, body weight at the 12^th^ week, FCR, feed conversion ratio; Seq, High-throughput sequencing markers; Chip, Chip markers.

As shown in **Table 6**, with marker screening by the GWAS method, the accuracy of genomic prediction for the two traits was also improved, but the gains of accuracy were less than the gains with PMS method for all traits. When using SLAF-seq, the accuracy of genomic prediction was increased by 0.098 for BW and 0.068 for FCR, and the gains were significant (P < 0.05). When using the SNP chip, the accuracy of genomic prediction was increased by 0.083 for BW and 0.020 for FCR and the gains were also significant (P < 0.05).

## Discussion

In this study, the genomic prediction of growth and feed efficiency traits in a small broiler chicken population was compared between high-throughput sequencing and SNP chip platforms. The results showed that when markers were not screened, the use of high-throughput sequencing data did not result in a higher accuracy than the use of chip data. Our method for marker screening, improved the accuracy of genomic prediction for both genotyping platforms, and high-throughput sequencing achieved higher accuracy for both traits.

With the rapid decline in the price of high-throughput sequencing, its application in genomic selection has received a high level of attention (Meuwissen and Goddard, 2010; Gorjanc et al., 2015; Iheshiulor et al., 2016). Meuwissen and Goddard (2010) used simulation data to study the accuracy of genomic selection based on high-throughput sequencing. The results showed that when using sequencing data, the accuracies of prediction of genetic values were 40% increased relative to the use of dense 30K SNP chips. Iheshiulor et al. (2016) showed that the accuracy of genomic selection using sequencing data can be increased by up to 92% in a simulation study. However, when using real data, researchers can hardly achieve such attractive results (Heidaritabar et al., 2016; Ni et al., 2017; Elbasyoni et al., 2018). In plant breeding, Elbasyoni et al. (2018) studied four traits in a winter wheat population and showed that high-throughput sequencing could achieve only comparable or even better accuracy than an SNP chip. In a commercial brown layer line, Ni et al. (2017) compared genomic predictions for three egg-laying traits using genome-wide sequencing and a 336K SNP chip and reported that little or no benefit was gained when using all sequencing SNPs for genomic prediction.

To improve the accuracy of genomic prediction, some previous studies tried to add causative mutations to chip data (Meuwissen and Goddard, 2010; Brøndum et al., 2015; Teissier et al., 2018). Many studies investigated the effect on the reliability of genomic prediction when a small number of important variants obtained from single marker GWAS or SNP annotation based on whole genome sequence data were added to the regular 54K SNP chip data (Van Binsbergen et al., 2015; Ma et al., 2019). In the current study, the SNP markers obtained by sequencing were twice as abundant as those of the SNP chip, but the prediction accuracy was not increased when all of the markers were used for genomic prediction. One of possible reasons is that the sequencing data increase the number of associated markers but also contribute a large number of unimportant markers that are not related to the traits of interest, which may interfere with the estimation of breeding values. Therefore, we proposed a new method to select effective markers; using our method for screening markers, the sequencing data had higher accuracy of genomic prediction than the SNP chip data for all traits.

The SNP markers farther from the causative mutations may negatively affect the accuracy of genomic prediction. As mentioned above, many previous studies (Zhang et al., 2014; Zhang et al., 2015; Van Den Berg et al., 2016; Ye et al., 2019) have shown that preselected markers from genomic data can improve genomic prediction. However, Ye et al. also performed genomic prediction using markers preselected from imputed whole-genome sequencing (WGS) data based on the *p* value of GWAS as a control method, and the results showed that using preselected variants resulted in almost no increase for most traits and even increased the bias of the predictions (Ye et al., 2019). The authors argued that one of the possible reasons could be the difficulty of detecting causal variants based on GWAS due to the large number of variants and the high LD between variants. In our study, we provided a method to select SNP markers based on the difference between phenotypes associated with two allelic homozygous genotypes, GWAS method to select markers was also performed as a comparison. Our results showed that when screening markers with the two methods, the accuracies of genomic prediction for the two traits were improved, and the gains of accuracy with PMS method were larger than the gains with GWAS method for all traits. Whether the PMS method can improve the accuracy of genomic prediction in different populations needs further verification.

The cost of genotyping is an important factor limiting its application in poultry breeding. With the continuous development of sequencing technology, the reduced price of sequencing may have advantages in regard to the cost of genotyping. De Donato et al. showed that the cost of sequencing data for the same number of markers is approximately 1/3 that of the SNP chip (De Donato et al., 2013). Among the major livestock and poultry breeds, the chicken genome is more advantageous with regard to the cost of sequencing. The genome of the chicken is only 1043.19 Mb (https://www.ncbi.nlm.nih.gov/genome), which is less than 1/2 the size of the genomes of cattle (2716 Mb) and pigs (2548 Mb), which means that chickens can achieve the same density of genome coverage at a lower cost. In addition, sequencing technology can flexibly select the depth of sequencing for a single sample, which can further reduce the cost of genotyping.

Local breeds usually have characteristic traits that are preferred by local people, allow the birds to adapt well to the local environment, and usually exist in the form of small populations with a small effective population size. Druet et al. showed that the accuracy of genomic prediction depends largely on the coverage of key genes affecting target traits by genotyping platforms (Druet et al., 2014). However, the markers of conventional SNP chips may not cover all of the genetic variation of specific traits in the local breeds, which may limit the efficiency of genomic prediction for these traits. High-throughput sequencing, such as SLAF-seq, can improve the accuracy of genomic prediction by optimizing the selection of suitable restriction enzymes to cover large fragments of specific mutation regions for local breeds and to select the sequencing depth of each individual, which has great potential for genomic prediction in local breeds breeding.

## Conclusions

The results from this study indicate that with our PMS marker screening method, the accuracy of genomic prediction obtained using high-throughput sequencing, such as SLAF-seq, is higher than the accuracy obtained using SNP chips in local broiler populations. With accurate prediction and a low cost, the PMS method is a promising method for the use of high-throughput sequencing data for genomic prediction in breeding programmes of local broiler populations.

## Data Availability Statement

Genotype data and trait data for the chickens used in this study are not publicly available, but are available from the corresponding author upon reasonable request.

## Ethics Statement

This study was approved by the Animal Care Committee of the Institute of Animal Science, Guangdong Academy of Agricultural Sciences (Guangzhou, People’s Republic of China)(No. GAAS-IAS2009-73).

## Author Contributions

TL, CL, and HQ conceived and designed the experiments. TL, CL, JM, YW, DS, GS, and HQ discussed and interpreted the results. TL drafted the manuscript. CL, JM, YW, DS, GS, and HQ revised the manuscript. All authors read and approved the final manuscript.

## Funding

This work was supported by the Earmarked Fund for Modern Agro-Industry Technology Research System (CARS-41), the Science and Technology Program of Guangdong (2017B020201006, 2017B020206003) and Key-Area Research and Development Program of Guangdong Province (2018B020203001). The funders had no role in study design, data collection and analysis.

## Conflict of Interest

The authors declare that the research was conducted in the absence of any commercial or financial relationships that could be construed as a potential conflict of interest.
